# Bacterial identification of the vaginal microbiota in Ecuadorian pregnant teenagers: an exploratory analysis

**DOI:** 10.7717/peerj.4317

**Published:** 2018-02-21

**Authors:** Ana María Salinas, Verónica Gabriela Osorio, Pablo Francisco Endara, Eduardo Ramiro Salazar, Gabriela Piedad Vasco, Sandra Guadalupe Vivero, Antonio Machado

**Affiliations:** 1 Instituto de Microbiología, Colegio de Ciencias Biológicas y Ambientales, Universidad San Francisco de Quito, Quito, Ecuador; 2 Colegio de Ciencias de la Salud, Universidad San Francisco de Quito, Universidad San Francisco de Quito, Quito, Ecuador; 3 Facultad de Ciencias Médicas, Universidad Central del Ecuador, Quito, Ecuador

**Keywords:** *Lactobacillus* sp., Pregnancy, Species identification, Vaginal microbiota, Bacterial vaginosis, 16S rRNA, Teenagers, Ecuador, 23S rRNA, Anaerobes

## Abstract

**Background:**

Bacterial vaginosis (BV) is a microbial imbalance (i.e., dysbiosis) that can produce serious medical effects in women at childbearing age. Little is known, however, about the incidence of BV or vaginal microbiota dysbiosis in pregnant teenagers in low and middle-income countries such as Ecuador. The scope of this exploratory analysis was to study the relationship between epidemiologic and microbial risk factors. Among the microbiology risk factors this study investigated five *Lactobacillus* species, two of them know in preview studies as microbiology risk factors for BV development (*Lactobacillus acidophilus* and *Lactobacillus iners*), and the last three known for being associated with a healthy vaginal tract (*Lactobacillus crispatus*, *Lactobacillus gasseri* and *Lactobacillus jensenii*). In addition, fastidious anaerobes known to be microbial risk factors for BV development in pregnant teenagers were searched as well, more exactly, *Gardnerella vaginalis*, *Atopobium vaginae* and *Mobiluncus mulieris*.

**Methods:**

Ninety-five healthy adolescent pregnant women, visiting a secondary level hospital in Quito, Ecuador, were enrolled into the study in 2015. The enrolled patients were between 10 to 13 weeks of pregnancy. Four epidemiological risk factors were collected in a survey: age, civil status, sexual partners and condom use. Also, vaginal pH was measured as a health risk factor. DNA was extracted from endocervical and exocervical epithelia from all the patients’ samples. PCR analysis was performed in order to characterize the presence of the eight bacterial species known as risk factors for BV development, targeting three anaerobes and five *Lactobacillus* species. Univariate and multivariate analysis were performed to identify associated factors for the presence of anaerobic species using logistic regression.

**Results:**

The 95 vaginal microflora samples of these teenagers were analyzed. Two of the bacterial species known to cause BV: *A. vaginae* (100%) and *G. vaginalis* (93.7%) were found in high prevalence. Moreover, the most predominant bacterial *Lactobacillus* species found in the pregnant teenagers’ vaginal tract were *L. crispatus* (92.6%), *L. iners* (89.5%) and *L. acidophilus* (87.4%). In addition, the average vaginal pH measured in the study population was 5.2, and high pH was associated with the presence of the three-anaerobic species (*p* = 0.001). Finally, *L. jensenii*’s presence in the study decreased in 72% the occupation of the three anaerobes.

**Discussion:**

This work identified a high pH as a risk factor for BV anaerobes’ presence in adolescent pregnant women. Moreover, this study identified *L. crispatus, L. iners* and *L. acidophilus* to be the most abundant species in our study population. From all fastidious anaerobes analyzed in this study, *A. vaginae* was present in all pregnant teenagers. To conclude, *L. jensenii* could be a potential healthy vaginal microbiota candidate in pregnant teenagers and should be further analyzed in future studies.

## Introduction

The bacterial microbiota of the vagina includes a diverse set of species (Ma, Forney & Ravel, 2012), consisting of a balance between anaerobic and aerobic microorganisms (Alvarez-Olmos et al., 2004). Commensal microorganisms in the vagina (vaginal microbiome) provide protection against opportunistic and pathogenic bacteria, constituting the first line of defense against invasive microorganisms (Ma, Forney & Ravel, 2012; Romero et al., 2014).

Several species of *Lactobacillus* sp. are dominant members of the vaginal commensal barrier and are usually associated with a healthy mucous membrane. Some of the species such as *L. crispatus*, *L. gasseri*, *L. jensenii* among others contribute in lowering pH through the production of great amounts of lactic acid and bactericide compounds (Wilks et al., 2004; Husain et al., 2014), such as H_2_O_2_ and bacteriocins (Wilks et al., 2004; Kiss et al., 2007; Tamrakar et al., 2007; Nelson et al., 2009; Machado et al., 2013; O’Hanlon, Moench & Cone, 2013; Romero et al., 2014). All these compounds and properties allow vaginal epithelial homeostasis and also keeping away possible pathogenic bacteria. However, not all *Lactobacillus* species found in the vaginal tract are strong probiotic species because of their low bactericide elements production such as *L. acidophilus* (Wilks et al., 2004), *L. iners* (Tamrakar et al., 2007) and others.

The vaginal microbiota of healthy women, during their reproductive age, is typically stable (Romero et al., 2014). When a woman is pregnant, however, hormone levels fluctuate, causing changes in the vaginal microbiota (Huang et al., 2014; Romero et al., 2014; MacIntyre et al., 2015). This fluctuation can be even more dramatic in teenagers, who may be at an increased risk for sexually transmitted diseases (STDs) and bacterial vaginosis (BV) (Alvarez-Olmos et al., 2004). In Ecuador, teenagers have a wide range of health care needs, in particular related to sexual and reproductive health, where the high rates of adolescent pregnancy and its health control are a national concern (Goicolea, 2010; Loaiza & Liang, 2013; Svanemyr et al., 2017).

BV is a condition in which the vaginal microbiota suffers a shift associated with a reduction in several species of probiotic *Lactobacillus* and an increase in the presence of anaerobes (*G. vaginalis*, *A. vaginae* and *Mobiluncus* sp.). BV also causes an individual to be more susceptible to STDs or suffer pre-term labor and late miscarriage (Wilks et al., 2004; Tamrakar et al., 2007; Nelson et al., 2009; Romero et al., 2014; McMillan et al., 2015).

The vaginal microbiota in pregnant teenagers has been characterized in certain studies, however, these studies are often conducted in high-income countries where hygiene conditions differ from low-income countries (Kiss et al., 2007; Nelson et al., 2009; Verstraelen et al., 2009; Aagaard et al., 2012; Huang et al., 2014; Husain et al., 2014; Hyman et al., 2014; Romero et al., 2014; MacIntyre et al., 2015). There are few studies that have been conducted in low- and middle-income countries (LMICs) such as Ecuador (Vaca et al., 2010) or Brazil (Ferreira et al., 2015), yet those analyses relied only on Nugent scores (gram stained smears). This study was therefore conducted to assess the presence of five *Lactobacillus* sp. (*L. crispatus*, *L. gasseri*, *L. jensenii*, *L. acidophilus* and *L. iners*) and three bacterial anaerobes (*A. vaginae*, *G. vaginalis* and *M. mulieris*) associated with BV in pregnant teenagers from Ecuador using PCR amplification of 16S and 23S rRNA genes.

## Materials and Methods

### Study area, design and subject selection

The study was carried out in a secondary level public hospital in Quito, Ecuador that serves as a national reference hospital and teaching hospital, which provides care to pregnant women, including family planning and postpartum support services (Moya, 2001). The hospital also provides services for pregnant teenagers (most of them from Hispanic ethnicity), and in 2014, 2,230 pregnant teens received assistance (Ministerio de Salud Pública del Ecuador, 2014).

From September 2015 to November 2015, 95 pregnant teenage women (10–19 years old) were enrolled into the study. The study population involved teenagers between 10 to 13 weeks pregnant and reported to have not taken antimicrobials for the previous 3 months. The enrolled patients were interviewed in a private room and survey included demographic and health questions, including: age, marital status, number of sexual partners in the last year and the use of preservatives (condoms) during intercourse. Applicants were excluded from the study if they reported having sexual intercourse within the last 48 h, or in case there was any evidence of macroscopic cervical bleeding or placenta and fetal disease. This investigation adopted a cross-sectional study design to determine the association between the presence of *Lactobacillus* sp. and BV related anaerobes.

### Ethics statement

Ninety-five volunteers met the eligibility criteria and read and signed the informed consent (if 18 or older) or had their parents or legal representative sign if they were less of 18 years old. The Ethics Committee of the Central University of Ecuador approved this study (Protocol code: cif-cv-fcm.1).

### Samples collection

Vaginal samples were collected by trained gynecologists. The procedure began by removing the cervical mucus with a sterile swab. Furthermore, endocervical and exocervical epithelia were gathered with a cervix examination brush (Rovers Cervex-Brush; Rovers Medical Devices B.V., Oss, The Netherlands). Additionally, the pH of the mucus samples was determined from the vaginal swab using a pH test strip (MColorpHast; Merck-Millipore, Burlington, MA, USA). The detachable head of the cervix examination brush was preserved in a liquid vial (BD SurePath™ liquid-based Pap test; BD Biosciences, San Jose, CA, USA) and transported under refrigeration conditions (4 °C). The samples were stored at −40 °C for 5 h until DNA extraction.

### DNA extraction

DNA from the vaginal brush was extracted by following commercial kit instructions (NucleoSpin^®^ Tissue 740952; Macherey-Nagel™, Düren, Germany) (Macherey-Nagel, 2014). Briefly, the brush samples were placed in 1.5 ml microtubes with 180 µl Buffer T1 and 25 µl Proteinase K. Then, to homogenate the mixture, the microtubes were centrifuged for 15 s at 1,500 × *g* and heated at room temperature for 5 min. Next, the tubes were vortexed vigorously for 15 s and span for 15 s at 1,500 × *g*. The microtubes were then incubated for 10 min at 70 °C, and followed by 5 min at 95 °C. The tubes were centrifuged again for 15 s at 1,500 × *g,* after which 200 µl of Buffer B3 was added. Tubes were vortexed vigorously for 30 s and heated at 70 °C for 10 min. Ethanol (96–100%) was added to each sample and vigorously vortexed once more. A Nucleospin Tissue Column was placed into a collection tube and the sample was added to the column. The tubes were then centrifuged for 1min at 11,000 × *g* and discarded and new ones were placed under the column. Kit buffers (BW and B5) were added to the columns and centrifuged for 1min at 11,000 × *g* for each buffer. The columns were dried with another centrifugation and finally DNA was eluted with buffer (BE) (Macherey-Nagel, 2014). The concentration of the extracted DNA was measured in a NANOVUE spectrophotometer (GE Healthcare Life Sciences, Little Chalfont, UK) to determine the DNA extraction quality. The original DNA extraction was then divided into two diluted aliquots with Buffer BE to a final concentration of 20 ng/µL. Finally, the remaining original samples were preserved at −80 °C, as well as the two aliquots of 20 ng/µL, were preserved at −20 °C for Polymerase Chain Reaction (PCR) analysis.

### Polymerase chain reaction

PCR amplification was performed for nine different primer sets, targeting three anaerobes and five *Lactobacillus* species and one primer set for *Lactobacillus* genus of vaginal microbiota (see Table 1). Samples of *L. jensenii* and *A. vaginae* were sequenced to confirm their identity due to the lack of a strong specificity, while *L. acidophilus*, *G. vaginalis* and *M. mulieris* primer sets showed a strong specificity and thus samples did not require a further sequencing confirmation. The eventual confirmation step used the following universal primers for 16S rRNA sequencing (27Fw-AGA GTT TGA TCM TGG CTC AG and 805Rw-GAC TAC CAG GGT ATC TAA TC; temperature of annealing: 62 °C; Tanner et al., 1999) through a PCR assay carried out with a final volume of 50-µl (adapted from the procedure below) and sent to Functional Biosciences, Inc (Madison, WI, USA). The 16S rDNA sequences were compared to known sequences in GenBank with the advanced gapped BLAST (basic local alignment search tool) algorithm (Camacho et al., 2009).

**Table 1:** Primers used in this study.

Set	Name	Sequence (5′–3′)	Target	T (°C) of annealing	Size of fragment	Target	Specificity %	Cross reaction with microorganisms	Validation	Reference
1	LactoF	TGGAAACAGRTGCTAATACCG	*Lactobacillus* spp.	55 °C	233 bp	16S rRNA	100%	Negative	Samples sequenced to confirm identity	Byun et al. (2004), Zhang et al. (2012)
	LactoR	GTCCATTGTGGAAGATTCCC								
2	LinersF	GTCTGCCTTGAAGATCGG	*L. iners*	55 °C	158 bp	16S rRNA	100%	Negative	Samples sequenced to confirm identity	De Backer et al. (2007), Zhang et al. (2012)
	LinersR	ACAGTTGATAGGCATCATC								
3	LcrispatusF	TTACTTCGGTAATGACGTTA	*L. crispatus*	55 °C	966 bp	16S rRNA	100%	Negative	Samples sequenced to confirm identity	De Backer et al. (2007), Zhang et al. (2012)
	LcrispatusR	GGAACTTTGTATCTCTACAA								
4	LgasseriF	TCGAGCGAGCTTGCCTAGATGAA	*L. gasseri*	60 °C	372 bp	16S rRNA	100%	n/d	Samples sequenced to confirm identity	Zhang et al. (2012)
	LgasseriR	CGCGGCGTTGCTCCATCAGA								
5	LjenseniiF	AGTCGAGCGAGCTTGCCTATAGAAG	*L. jensenii*	57 °C	342 bp	16S rRNA	100%	Negative	Samples sequenced to confirm identity in this study	Garg et al. (2009), Zhang et al. (2012)
	LactoR	GTCCATTGTGGAAGATTCCC								
6	LA-1 Fw	TCAATCAAAGGAAGACGCAG	*L. acidophilus*	56 °C	221 bp	23S rRNA	100%	Negative	n/d	Tsai et al. (2010)
	LA-2 Rw	CGCTCGCAATTTCGCTTA								
7	Gard154-Fw	CTCTTGGAAACGGGTGGTAA	*Gardnerella vaginalis*	60 °C	301 bp	16S rRNA	100%	Negative	n/d	Henriques et al. (2012)
	Gard154-Rv	TTGCTCCCAATCAAAAGCGGT								
8	Mobil-577F	GCTCGTAGGTGGTTCGTCGC	*Mobiluncus mulieris*	62 °C	449 bp	16S rRNA	100%	Negative	n/d	Fredricks et al. (2007)
	M.mulie-1026R	CCACACCATCTCTGGCATG								
9	Atop109-Fw	GAGTAACACGTGGGCAACCT	*Atopobium vaginae*	62 °C	221 bp	16S rRNA	16.7%	5 from 6	Samples sequenced to confirm identity in this study	Henriques et al. (2012)
	Atop109-Rv	CCGTGTCTCAGTCCCAATCT					37.5%	15 from 24		

**Notes.**

N/d, non determined.

The PCR assays were performed with T100™ thermal cycler (Bio-Rad Laboratories, Hercules, CA, USA) in a reaction volume of 20 µL. The reaction mix included 4 µL 5X Green GoTaq^®^ Flexi Buffer (Promega, Madison, WI, USA), 2 µL MgCl_2_ 2.5 mM (Promega, Madison, WI, USA), 0.50 µL dNTP mix 10 mM (Promega, Madison, WI, USA), 0.20 µL GoTaq^®^ Flexi DNA Polymerase (Promega, Madison, WI, USA), 1.0 µL of each primer 0.5 µM, 2 µL from each DNA sample and the remaining volume with molecular grade H_2_O.

PCR amplification first cycle consisted in a pre-melt phase at 94 °C for 2 min and then denaturation at 94 °C for 30 s. After that, annealing at each species temperature (see Table 1) was realized for 30 s, and extension was performed at 72 °C for 1 min. This was repeated for 29 more cycles to 31 for the bigger amplicons (>500 bp). An additional 5 min extension was included at the end of the cycles to complete the extension of the primers. The PCR products were visualized using electrophoresis in 2% agarose gels and staining with ethidium bromide 0.1%, with negative and positive controls provided by the Microbiology Institute at Universidad San Francisco de Quito.

### Statistical analysis

Uni- and multivariate analysis were realized in order to identify risk factors such as: age; civil status; number of sexual partners; condom use; vaginal pH; and *Lactobacillus* spp. associated with the presence or absence of anaerobic species (i.e., outcome) using logistic regression. Most variables were treated as categorical. For testing differences in the studied factors and vaginal microbiota between the groups of patients, the study was defined by the presence of three anaerobic species (*A. vaginae*, *G. vaginalis* and *M. mulieris*). Chi-square statistics were computed and logistic regression was used to estimate odds ratios for association between studied factors on the presence of three anaerobic species (*A. vaginae*, *G. vaginalis* and *M. mulieris*). Statistically significant differences were assumed when *P*-values were equal or less than 0.05. Statistical analyses were performed using STATA version 14.0 (StataCorp, College Station, TX, USA).

## Results

### Characteristics of study population

Most of the study participants were between 16–17 years old (53.7%) and non-married (68.4%). Approximately two thirds had only one sexual partner (76.8%) and around half did not use condoms (52.6%). In addition, all enrolled participants were from Hispanic ethnicity and nearly one third of the study population had a vaginal pH higher than five (31.5%). The pH mean value found in this study group was of 5.2 with a standard deviation (SD) of 0.7 and an interval of 4–7, Table 2 provides descriptive statistics of the study population.

**Table 2:** Epidemiological characteristics of pregnant teenagers in this study.

Epidemiological data		
	N°	%
**Age**		
12 to 15 years	18	19.0
16 to 17 years	51	53.7
18 to 19 years	26	27.4
**Civil status**		
Single	65	68.4
Free union	29	30.5
Married	1	1.1
**Sexual partners**		
One	73	76.8
More than one	22	23.2
**Condom use**		
No	50	52.6
Sometimes (Occasionally, yes)	45	47.4
**Vaginal pH**		
4 to 5	63	68.5
6 to 7	29	31.5

### Commensal microbiota

All samples showed the presence of *Lactobacillus* spp. The most frequently detected were *L. crispatus* 88/95 (92.6%), *L. iners* 85/95 (89.5%) and *L. acidophilus* 83/95 (87.4%). While *L. jensenii* and *L. gasseri* were only found in 77/95 (81.1%) and 54/95 (56.8%) of the studied pregnant teenagers, respectively.

**Figure 1:**
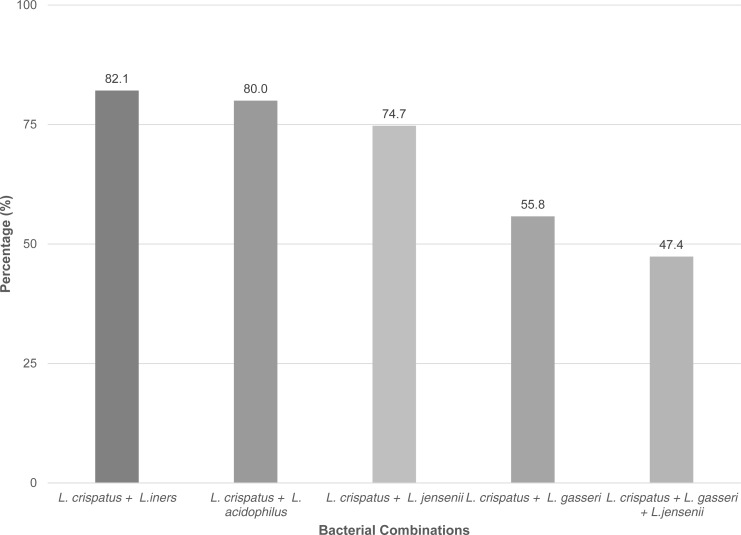
Percentage of grouped *Lactobacillus* species found between the samples.

All study participants were colonized by at least two of the five *Lactobacillus* species, which were analyzed. The most frequent combination was *L. crispatus* and *L. iners* in 82.1%. *L. crispatus* and *L. acidophilus*, were the second most prevalent combination with 80.0%, respectively. Other types of combinations were less frequent: *L. crispatus and L. jensenii* (74.7%); *L. crispatus* and *L. gasseri* (55.8%); and *L. crispatus*, *L. gasseri* and *L. jensenii* (47.4%), as shown in Fig. 1.

### BV associated anaerobes

The main BV associated anaerobic bacteria found in our study population was *A. vaginae* (found in 100% of the samples) followed by *G. vaginalis* and *M. mulieris* (in 93.7% and 34.7% of the samples, respectively).

**Figure 2:**
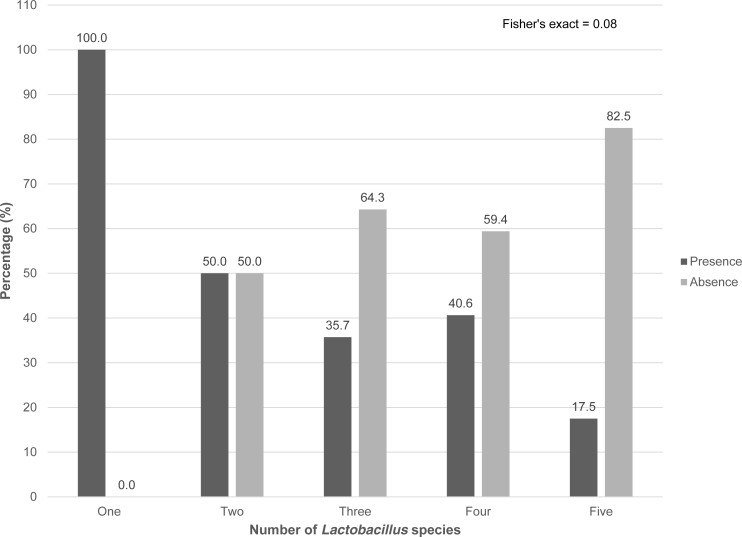
Percentage of teenagers with and without the presence of the three BV-associated anaerobes by the number of *Lactobacillus* species pre-existent in the vaginal epithelium. Presence or absence of three anaerobic species by number of *Lactobacillus* species.

When we looked at the presence of anaerobic bacteria in combination with the presence of *Lactobacillus* species, less than one-fifth of the samples had five *Lactobacillus* spp. and the three-anaerobic species (17.5%). Nevertheless, as showed in Fig. 2, no statistical significance was found for the association between the number of *Lactobacillus* species and the presence of three anaerobic species (*P*-value for Fisher’s exact test >0.05).

### Factors associated with presence of *Gardnerella vaginalis*, *Atopobium vaginae* and *Mobiluncus mulieris*

In univariate analysis, higher pH was statistically associated with the presence of three anaerobes. Having a pH higher than 5 increased the likelihood that three anaerobes would be present in the samples (crude OR 4.7, 95% CI [1.82–12.3]), as shown in Table 3. In contrast, the presence of *L. jensenii* in vaginal microbiota was associated with a decreased presence of three-anaerobic species (crude OR 0.28, 95% CI [0.1–0.81]), as shown in Table 4.

**Table 3:** Factors associated with the presence or absence of three anaerobic bacterial species (*Gardnerella vaginalis*, *Atopobium vaginae* and *Mobiluncus mulieris*) that are known to be associated with bacterial vaginosis.

	Absent	Present	*P*-value	OR	*P*-value
**Age**					
12 to 15 years	10 (15.4)	8 (26.7)		1	
16 to 17 years	34 (52.3)	17 (56.7)		0.62 (0.21–1.87)	0.400
18 to 19 years	21 (32.3)	5 (16.7)	0.21	0.3 (0.08–1.14)	0.078
**Civil status**					
Single	44 (67.7)	21 (70)		1	
Free union	20 (30.8)	9 (30)		0.94 (0.37–2.42)	0.900
Married	1 (1.5)	0 (0.0)	0.99	–	
**Condom use**					
No	33 (50.8)	17 (56.7)		1	
Sometimes (occasionally, yes)	32 (49.2)	13 (43.3)	0.59	0.79 (0.33–1.88)	0.593
**Sexual partners**					
One	48 (73.9)	25 (83.3)		1	
More than one	17 (26.1)	5 (16.7)	0.31	0.56 (0.19–1.71)	0.312
**pH**					
4 to 5	50 (79.4)	13 (44.8)		1	
6 to 7	13 (20.6)	16 (55.2)	0.001	4.7 (1.82–12.3)	**0.001**

**Table 4:** Association of *Lactobacillus* spp. with the presence or absence of three anaerobic bacterial species (*Gardnerella vaginalis*, *Atopobium vaginae* and *Mobiluncus mulieris*) that are known to be associated with bacterial vaginosis.

Bacterial species	Absent	Present	*P*-value	OR	*P*-value
***Lactobacillus* spp.**					
Absence	(0.0)	0 (0.0)		1	
Presence	65 (100.0)	30 (100.0)		does not apply	
***Lactobacillus iners***					
Absence	6 (9.2)	4 (13.3)		1	
Presence	59 (90.8)	26 (86.7)	0.54	0.66 (0.17–2.5)	0.547
***Lactobacillus crispatus***					
Absence	4 (6.1)	3 (10)		1	
Presence	61 (93.9)	27 (90)	0.51	0.59 (0.12–2.8)	0.509
***Lactobacillus gasseri***					
Absence	25 (38.5)	16 (53.3)		1	
Presence	40 (61.5)	14 (46.7)	0.17	0.55 (0.23–1.3)	0.176
***Lactobacillus jensenii***					
Absence	8 (12.3)	10 (33.3)		1	
Presence	57 (87.7)	20 (66.6)	0.01	0.28 (0.1–0.81)	**0.02**
***Lactobacillus acidophilus***					
Absence	6 (9.2)	6 (20)		1	
Presence	59 (90.8)	24 (80)	0.14	0.41 (0.12–1.38)	0.151

**Notes.**

women/state; Nd, Not detected.

When the associations (Table 3) were adjusted for age, condom use and number of sexual partners, the only significant factor associated with presence of three anaerobes was a high pH (adjusted OR = 5.5; 95% CI [2–15.2], *P*-value: 0.002). After adjusting for the same variables in Table 4, the presence of *L. jensenii* remained to be associated with the absence of three anaerobes (adjusted OR 0.16; 95% CI [0.04–0.6], *P*-value: 0.006).

## Discussion

In the current study, we found that the genital tract of pregnant Ecuadorian teenagers was colonized primarily by *L. crispatus*. All samples from the studied teenagers were positive for at least one analyzed *Lactobacillus* species. Additionally, from the five-species analyzed in this study, colonization by the consortium formed through *L. crispatus*, *L. iners* and *L. acidophilus* was the most prevalent. These results coincide with previous reports indicating that *Lactobacillus* species is the most abundant commensal bacteria found in healthy vaginal microbiota of pregnant and non-pregnant adult women in the world, as shown in Table 5 (Kiss et al., 2007; Tamrakar et al., 2007; Verstraelen et al., 2009; Dominguez-Bello et al., 2010; Hernández-Rodríguez et al., 2011; Aagaard et al., 2012; Husain et al., 2014; Hyman et al., 2014; Romero et al., 2014; MacIntyre et al., 2015; McMillan et al., 2015). However, it is important to mention that other *Lactobacillus* species could have been present on the vaginal epithelium of these pregnant teenagers because this study did not analyze every *Lactobacillus* species but only the most frequently cited in other studies (see Table 5). We found a large proportion of individuals colonized by BV associated anaerobic bacteria, including: *A. vaginae* (100%), and *G. vaginalis* (93.7%), which showed some discrepancies with previous studies carried out in adult women (Table 5). The percentage of *A. vaginae* in this study was considerably different from other published studies due to the presence of *A. vaginae* in 100% of the analyzed pregnant teenagers in Ecuador. In agreement with the previous results, *G. vaginalis* was also found in more than 93% of the pregnant teenagers. As referenced by previous studies, *G. vaginalis* and *A. vaginae* are the main anaerobes associated with BV (Menard et al., 2008; Verstraelen & Swidsinski, 2013; Bretelle et al., 2015; Machado & Cerca, 2015; Hardy et al., 2016).

**Table 5:** Summary of vaginal microbiota characterization studies in pregnant women (including this study).

N°	Population description	Study group (*n*)	Place	Methodology	pH (mean)	Bacterial species detected (%)										Author
						*Lactobacillus* sp.	*L. iners*	*L. crispatus*	*L. acidophilus*	*L. jensenii*	*L. gasseri*	*G. vaginalis*	*A. vaginae*	*M. mulieris*	Other *Lactobacillus* sp.	
1	Pregnant women (Age range 18–35)	126	Austria	Multiplex PCR by Culture	Nd	57.1	Nd	19.8	Nd	11.9	Nd	Nd	Nd	Nd	YES	Kiss et al. (2007)
2	Pregnant women (Age range Nd)	100	Belgium	tRFLP—PCR	Nd	X	40.3	23.4	Nd	3.9	40.3	Nd	Nd	Nd	NO	Verstraelen et al. (2009)
3	Pregnant and non-pregnant women (Age range 19.4–39.2)	34	China	PCR and 16 sregion sequencing	4.5	X	55	26.9	Nd	Nd	6.3	11.8	11.8	Nd	NO	Huang et al. (2014)
4	Pregnant teenagers (Age range 12–19)	95	Ecuador	PCR Electrophoresis gel	5.2	100	89.5	92.6	87,4	48.4	56.8	93.7	100	34.7	NO	This study (2018)
5	Pregnant women (Age range 19–44)	132	Japan	PCR Culture	Nd	98.5	41.7	51.5	Nd	25	31.8	Nd	Nd	Nd	NO	Tamrakar et al. (2007)
6	Pregnant women (Age range 13–43)	140	Mexico	PCR (DGGE)	Nd	98.4	56.7	Nd	78.1	Nd	20.3	Nd	Nd	2.0	YES	Hernández-Rodríguez et al. (2011)
7	Pregnant and non-pregnant women (Age range 18–55)	131	Rwanda	16s region pyrosequencing	4.6	X	X	X	Nd	X	X	X	X	Nd	NO	McMillan et al. (2015)
8	Pregnant women (Age range Nd)	42	United Kingdom	16s region pyrosequencing	Nd	X	30^a^	43^a^	Nd	14^a^	9^a^	Nd	2^a^	Nd	YES	MacIntyre et al. (2015)
9	Pregnant women (Age range 28–31)	293	United Kingdom	Culture MALDI-TOF	Nd	75	Nd	37	Nd	45	34	Nd	Nd	Nd	YES	Husain et al. (2014)
10	Pregnant and non-pregnant women (Age range Nd)	54	USA	16s region pyrosequencing	Nd	X	58.5^a^	38.1^a^	Nd	X	4.3^a^	2.2^a^	2.2^a^	Nd	YES	Romero et al. (2014)
11	Pregnant and non-pregnant women (Age mean 31.4)	84	USA	16s region pyrosequencing	Nd	X	X	X	Nd	X	Nd	Nd	Nd	Nd	YES	Aagaard et al. (2012)
12	Pregnant women (Age ≥18)	88	USA	PCR Sanger sequencing	Nd	X	76.9 African 21.7 Asian 23.1 Caucasian 68.4 Hispanic	30.8 African 41.5 Caucasian 15.8 Hispanic	21.7 Asian 1.5 Caucasian	7.7 African 27.7 Caucasian 31.6 Hispanic	7.7 African 17.4 Asian 41.5 Caucasian 10.5 Hispanic	Nd	X	Nd	YES	Hyman et al. (2014)
13	Pregnant women (Age mean 24)	50	USA	qPCR	Nd	Nd	Nd	X	Nd	Nd	Nd	66.7	Nd	Nd	NO	Nelson et al. (2009)
14	Pregnant women (Age range 21–33)	9	Venezuela	PCR yrosequencing	Nd	88–94	Nd	Nd	Nd	Nd	Nd	0.1–2	4–34	Nd	NO	Dominguez-Bello et al. (2010)

**Notes.**

Legend: X, species analyzed but no percentage reported; Nd, Not detected.

a Results only from pregnant women/state.

The association between having a certain commensal vaginal microbiota with a healthy pregnancy outcome has been thoroughly studied in the past two decades (Kiss et al., 2007; Nelson et al., 2009; Verstraelen & Swidsinski, 2013). It has been postulated that an optimal commensal microbiota in the vaginal epithelium reduces the risk of infections with any pathogen (*Trichomonas vaginalis*, *Neisseria gonorrhoeae*, *Chlamydia trachomatis*, *Candida* sp., and others) in the reproductive track and also seems to prevent preterm birth caused by bacterial vaginosis (O’Hanlon, Moench & Cone, 2013; Krauss-Silva et al., 2014). Several studies around the world (see Table 5), such as in USA (Hyman et al., 2014; Romero et al., 2014), United Kingdom (MacIntyre et al., 2015), Belgium (Verstraelen et al., 2009), China (Huang et al., 2014) and Japan (Tamrakar et al., 2007), showed that the most abundant *Lactobacillus* species found in pregnant women are *L. iners* and *L. crispatus*. In agreement to this study, these two species were the most abundant lactobacilli found in pregnant teenagers in Ecuador. The third most abundant *Lactobacillus* species found in this study was *L. acidophilus*, which has only been observed in studies from Mexico (Hernández-Rodríguez et al., 2011) and USA (Hyman et al., 2014). In USA, it has been encountered in non-pregnant teenager’s vaginal microbiota with a prevalence of 49% (Alvarez-Olmos et al., 2004). *L. acidophilus* has not been analyzed or encountered in other studies worldwide as shown in Table 5.

Other *Lactobacillus* species, such as *L. jensenii* and *L. gasseri*, are usually found in high percentages in other studies of vaginal microbiota present in pregnant women (Tamrakar et al., 2007; Verstraelen et al., 2009; Husain et al., 2014; Hyman et al., 2014; MacIntyre et al., 2015). However, *Lactobacillus* species, such as *L. delbruecki, L. rhamosus, L. reuteri, L. casei. L. paracasei, L. vaginalis, L. coleohominis, L. fermentum, L. fornicalis, L. gallinarum, L. helveticus, L. kefiranofaciens, L. kitasatonis, and L. ultunensis,* have been analyzed as well but are not commonly found in studies of pregnant women’s vaginal microbiota (Hyman et al., 2014). Nonetheless, *L. jhonsonii* was detected by 16S rRNA pyrosequencing characterization of vaginal microbiota in pregnant women in USA (Aagaard et al., 2012), although this species was not analyzed in the current study. Therefore, a more extensive analysis may be necessary in the future.

*Atopobium vaginae* was the most frequent anaerobe observed in vaginal microbiota from pregnant teenagers in our study (Table 5), and it has been detected in USA (Hyman et al., 2014; Romero et al., 2014), Mexico (Hernández-Rodríguez et al., 2011), China (Huang et al., 2014), Europe (Bretelle et al., 2015; MacIntyre et al., 2015) and Africa (McMillan et al., 2015). This anaerobe is usually found in association with *G. vaginalis* and *M. mulieris* (Hernández-Rodríguez et al., 2011) in the development of BV (Verstraelen & Swidsinski, 2013). As similar as *G. vaginalis*, *A. vaginae* is usually found in minor percentages around the world (Nelson et al., 2009; Dominguez-Bello et al., 2010; Huang et al., 2014; Romero et al., 2014; MacIntyre et al., 2015). The percentage of *A. vaginae* in this study was markedly different from other published studies. In our study, *A. vaginae* was present in 100% of the analyzed pregnant teenagers in Ecuador. In agreement with previous results, *G. vaginalis* was also found in more than 93% of pregnant teenagers. Although this study did not quantify the abundance of *G. vaginalis* or *A. vaginae*, the prevalence of these anaerobes is not concordant with the normal prevalence in healthy pregnant women in other studies (Nelson et al., 2009; Dominguez-Bello et al., 2010; Huang et al., 2014; Romero et al., 2014), such as Africa where 66.7% of healthy pregnant women had *G. vaginalis* (Nelson et al., 2009) or the USA where 2.2% of healthy pregnant women had *G. vaginalis* or *A. vaginae* (Romero et al., 2014), which is low in comparison with the results from this study. Finally, only 34.5% of the pregnant teenagers were positive for *M. mulieris,* which is high in comparison to other studies performed in pregnant women such as 2.0% in Mexico (Hernández-Rodríguez et al., 2011), 0.37% in Rwanda (Tchelougou et al., 2013) and 18.2% in USA (Waters et al., 2008).

The major drawback of this study was the lack of quantitative data which may allow us to assess the status of the colonization of the distinct bacterial taxa*.* However, BV is a very common cause for high pH in the vagina, associated with the shifting of microbiota from protective *Lactobacillus* sp. to anaerobes, such as *G. vaginalis* and *A. vaginae* (De Backer et al., 2007). When this occurs, the pH rises from 4.2 that is considered normal to 4.5 or higher, which is now used as a positive indicator for BV (O’Hanlon, Moench & Cone, 2013). The pH in pregnant women has been analyzed in several studies, such as China (Huang et al., 2014) and Rwanda (McMillan et al., 2015) where the pH media was 4.5 and 4.6, respectively. In this study, however, the observed pH was 5.2, which is a marker for the shift in dominance from *Lactobacillus* to anaerobic microbiota on the vaginal epithelium. The presence of the three anaerobes analyzed in this study appeared to increase the vaginal pH, which is in agreement with other studies (McMillan et al., 2015; Donders et al., 2016) that evaluated the establishment of BV in women.

This exploratory study was able to characterize the heterogeneity of the commensal *Lactobacillus* species in these pregnant teenagers, where all study participants had more than one *Lactobacillus* species colonizing the vagina. As shown in Fig. 2, the reproductive track of our study participants revealed two to five *Lactobacillus* species. Even though there is no statistical significance with the absence or presence of the three anaerobes, our results differed from what was published in other studies (Hernández-Rodríguez et al., 2011; Hyman et al., 2014). In Mexico, it has been reported that the vagina was colonized by one to four species (Hernández-Rodríguez et al., 2011), meanwhile in the USA by one to two species (Hyman et al., 2014). *L. crispatus*, *L. gasseri* and *L. jensenii* (Wilks et al., 2004; Husain et al., 2014) are known as strong beneficial species, however *L. acidophilus* (Wilks et al., 2004), and *L. iners* (Tamrakar et al., 2007) are known as weaker protective species. Therefore, the high prevalence of these two species in the vaginal microbiota could partially explain the high prevalence of *A. vaginae* and *G. vaginalis* in Ecuadorian pregnant teenagers. Even without a statistical difference, the high prevalence of *L. iners* and *L. acidophilus* could imply some sort of bewilderment in the commensal bacteria of the vaginal microbiota, which could allow the proliferation of anaerobic bacteria. Further study is necessary to quantify each *Lactobacillus* species and anaerobic bacteria in each sample in order to determine the exact load of the detected vaginal microbiota in pregnant teenagers. Also, the amount of the beneficial substances produced by *Lactobacillus* species found in Ecuadorian women, such as H_2_O_2_ or lactic acid, should be quantified to determine their protective ability.

Finally, this study limitations were principally caused by the low number of samples, and the lack of bacterial abundance detection.

## Conclusion

Our study identified *L. crispatus, L. iners* and *L. acidophilus* to be the most abundant species in our study population of pregnant teenagers*. L. jensenii* could be a potential microbiota protective candidate in the vaginal microbiota in pregnant Ecuadorian teenagers. Meanwhile, *A. vaginae*, *G. vaginalis* and *M. mulieris* were found in 100%, 93.7% and 34.7% of the analyzed pregnant teenagers, respectively.

To the authors’ knowledge, this is the first study of vaginal microbiota in pregnant teenagers in Ecuador. Furthermore, this investigation was only realized in pregnant teenagers and there was no control group (non-pregnant teenagers). Further studies are necessary in pregnant women to quantify the main *Lactobacillus* species and also the BV associated anaerobes identified in this pilot study, as well as to statistically determine a possible correlation with preterm birth or BV in Ecuadorian pregnant women, which was not possible to characterize in this exploratory study.

##  Supplemental Information

10.7717/peerj.4317/supp-1 File S1 Raw data epidemiological file from this study

## List of abbreviations

BV: Bacterial vaginosis
STDs: Sexually Transmitted Diseases
LMICs: Low and Middle-Income Countries

## Additional Information and Declarations

### Data Availability

The following information was supplied regarding data availability: The raw data has been provided as a Supplemental File.
